# Could the eIF2α-Independent Translation Be the Achilles Heel of Cancer?

**DOI:** 10.3389/fonc.2015.00264

**Published:** 2015-11-26

**Authors:** Martin Holcik

**Affiliations:** ^1^Department of Pediatrics, Apoptosis Research Centre, Children’s Hospital of Eastern Ontario Research Institute, University of Ottawa, Ottawa, ON, Canada

**Keywords:** eIF2α, selective translation, IRES, stress, oncogene, apoptosis

## Abstract

Eukaryotic initiation factor eIF2 is a key component of the ternary complex whose role is to deliver initiator tRNA into the ribosome. A variety of stimuli, both physiologic and pathophysiologic activate eIF2 kinases that phosphorylate the α subunit of eIF2, preventing it from forming the ternary complex, thus attenuating cellular protein synthesis. Paradoxically, in cancer cells, the phosphorylation of eIF2α is associated with activation of survival pathways. This review explores the recently emerged novel mechanism of eIF2α-independent translation initiation. This mechanism, which appears to be shared by some RNA viruses and Internal Ribosome Entry Site-containing cellular mRNAs and utilizes auxiliary proteins, such as eIF5B, eIF2D, and MCT-1, is responsible for the selective translation of cancer-associated genes and could represent a weak point amenable to specific targeting for the treatment of cancer.

## Introduction

Regulation of protein synthesis (translation) is a key cellular process, which underpins cellular survival. Traditionally, the transcription and translation of the genome was considered to be a highly correlated phenomenon, this notion has been challenged by the demonstration that mRNA transcriptional outputs correlate with only about 40% of the total protein content in a cell (1, 2). This disconnect is further accentuated upon e.g., epidermal growth factor stimulation where up to 90% of transcripts exhibited uncoupled translation from transcription (2). Interestingly, these studies also showed that highly expressed transcripts are sometimes poorly translated and, *vice versa*, poorly transcribed genes can be translated efficiently. This suggests that transcription and translation are largely independent of each other, further strengthening the notion that translational control has a major impact on regulating the proteome under certain conditions. It is therefore not surprising that many aberrant cellular processes require modification of the translation machinery and translation output (i.e., proteome). This is often the case in various stress responses and diseases and is probably best exemplified in cancer. The link between misregulated translation and cancer has long been suspected, and strong experimental evidence has been obtained by many laboratories in recent years. This review will focus on one specific mechanism of translational dysregulation in oncogenesis, which is the ability of cancer cells to utilize an eIF2α-independent mode of translation initiation.

## eIF2 is the Nexus That Controls Translation Initiation Efficiency

**Figure 1:**
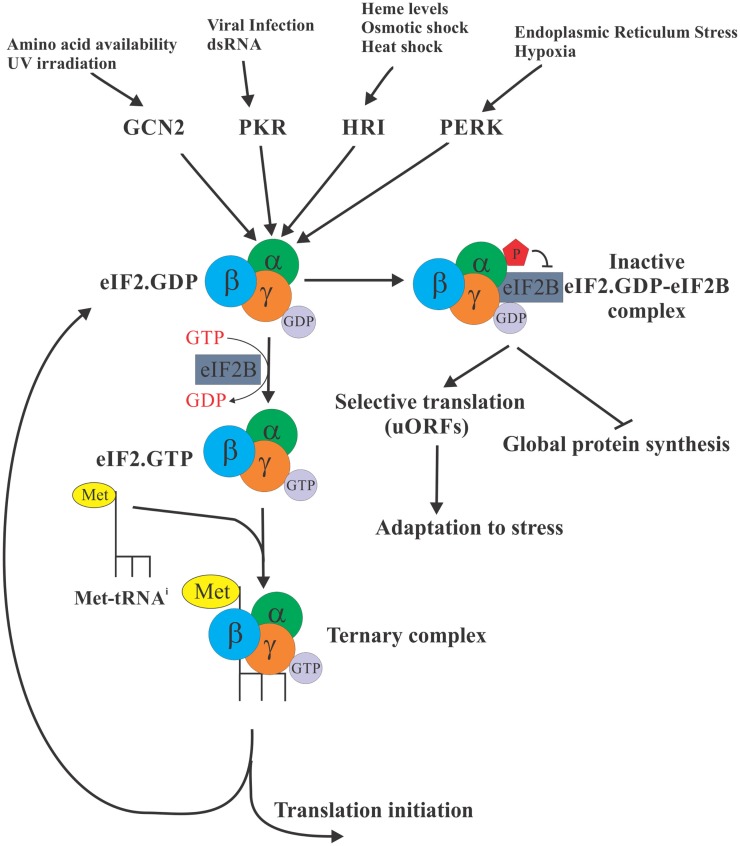
**eIF2α is a control nexus integrating diverse stimuli to regulate translation**. The eukaryotic initiation factor 2 (eIF2) is required for the formation of the ternary complex [consisting of eIF2 (α, β, and γ subunits), Met-tRNA_i_, and GTP] whose role is to deliver the initiator Met-tRNA_i_ to the P site of the 40S ribosomal subunit, enabling translation initiation. eIF2 exists in two distinct configurations – the GDP bound (inactive) and GTP bound (active), but only the GTP-bound form can bind Met-tRNA_i_. In response to diverse stress stimuli, distinct protein kinases phosphorylate the α subunit of eIF2 at serine 51, ultimately locking eIF2 in an inactive eIF2 complex with eIF2B. As a result, availability of the ternary complex significantly declines ultimately resulting in the inhibition of translation globally. Selective translation of a subset of mRNAs with uORFs continues, however, allowing cells to express proteins required for adaptation to stress conditions.

The eukaryotic initiation factor 2 (eIF2) is required for the formation of the ternary complex [consisting of eIF2 (α, β, and γ subunits), Met-tRNA_i_, and GTP], which in turn brings the initiator Met-tRNA_i_ to the P site of the 40S ribosomal subunit, enabling initiation of protein synthesis. eIF2 exists in two distinct configurations – the GDP- or GTP-bound forms. Following the recognition of the start codon by the 43S preinitiation complex, the GTP bound to eIF2 is hydrolyzed to deliver Met-tRNA_i_ and the eIF2. GDP is subsequently released from the 40S subunit to be recycled for the delivery of another Met-tRNA_i_ [reviewed in Ref. (3)]. The GDP to GTP exchange is catalyzed by the eIF2B (the guanine exchange factor). This step is necessary for the regeneration of active eIF2, which is the only form of eIF2 that can bind Met-tRNA_i_. Regulation of the GDP–GTP exchange, thus, provides the cell with a control point to match the protein synthesis rates to the physiologic requirements (Figure 1). In response to virtually all stresses, the α subunit of eIF2 is phosphorylated at serine 51, which greatly increases its avidity for eIF2B and locks both proteins in an inactive eIF2–eIF2B complex. Since eIF2B is present in limiting concentrations, phosphorylation of even a small fraction of eIF2α is sufficient to inhibit its recycling. As a result, availability of the ternary complex significantly declines leading to inhibition of 43S formation and attenuation of translation initiation (4).

In mammalian cells, there are four eIF2α kinases that integrate a variety of distinct stresses to phosphorylate eIF2α. Thus, haem-regulated inhibitor (HRI) is activated by iron deficiency (5), heavy metals (6), osmotic, or oxidative stress and heat shock (7); protein kinase activated by double-stranded RNA (PKR) is activated by dsRNA during viral infection (8) or interferon-induced apoptosis (9); general control non-derepressible-2 (GCN2) is activated by amino acid starvation (10) and UV irradiation (11); and PKR-like endoplasmic reticulum kinase (PERK) is activated in response to endoplasmic reticulum (ER) stress (12) and UV irradiation (13). Since the ternary complex is absolutely required for the translation of cellular mRNAs, the phosphorylation of eIF2α is expected to inhibit translation globally. However, for certain mRNAs, phosphorylation of eIF2α enhances their translation. These mRNAs frequently encode for proteins required for stress adaptation and recovery [reviewed in Ref. (14)]. These mRNAs, best exemplified by the transcription factor ATF4, contain short upstream ORFs (uORFs) in their 5′ untranslated regions (UTRs) that engage the ribosome and thus cause the main coding ORF of these mRNAs to be poorly translated when ternary complex is abundant. In contrast, during stress conditions when the abundance of ternary complex is limited, some ribosomes pass through the uORFs without initiating until they acquire a ternary complex upstream of the main coding ORF, resulting in productive translation of the main ORF. It is expected, however, that under conditions of severe stress if no ternary complex is available even translation of these mRNAs would be inhibited.

## Evidence for eIF2α-Independent Translation

In the last decade or so, evidence has begun to emerge pointing to the existence of an eIF2α-independent initiation of translation. It was observed in several independent studies that phosphorylation of eIF2α is frequently required to promote cell survival under various stresses, but the genes lying downstream of eIF2α regulation did not necessarily utilized the uORF-dependent mechanism. For example, following nutritional stress caused by amino acid starvation, induction of amino acid transporter SNAT2, a component of transport system A, which is required to promote recovery of amino acid balance, was blunted in cells unable to phosphorylate eIF2α (15). Similarly, translation of the inhibitor of apoptosis proteins XIAP and cIAP1 was shown to be resistant to the global attenuation of translation due to eIF2α phosphorylation during hypoxia (16, 17), glucose deficiency (18), serum deprivation (19–21), or ER stress (22). Other examples of mRNAs whose translation is enhanced by eIF2α phosphorylation include VEGF and p120 catenin [observed in hypoxic inflammatory breast tumors (23, 24), Bcl-xL [during hypertonic stress (25)], or c-Src and c-myc [during ER stress (26, 27)]. Interestingly, none of these mRNAs harbor uORFs that would enable enhanced mRNA translation under conditions of reduced ternary complex availability. Instead, Internal Ribosome Entry Site (IRES) sequences were described in the 5′ UTRs of these mRNAs. IRESs are discrete functional RNA motifs that support translation of the downstream reading frame by an alternative mechanism that does not appear to rely on m^7^G cap structure or initiation factors that recognize the cap. This phenomenon, termed internal initiation, was first observed in RNA viruses (in particular *Picornaviridae*) whose RNA is naturally uncapped, and yet, it is efficiently translated by the host translation machinery (28). Instead of cap, IRESs precede the protein-coding portion of the viral RNA and directly recruit the 40S ribosome to the vicinity of initiation codon. The recruitment of the 40S can occur either in the complete absence of any other protein factors (dicistrovirus intergenic IRES) or with the aid of various combinations of canonical initiation factors (such as eIF4G and eIF3) and auxiliary proteins in case of the remaining viral IRES [reviewed in Ref. (29)].

Similarly to the internal initiation in viruses, some cellular mRNAs were shown to be translated by this alternative mode of translation. It was observed that a subset of cellular mRNAs (up to 5%) was efficiently translated in cells infected with poliovirus which cleaves eIF4G and thus inhibits cap-dependent translation (30). Follow-up studies identified functional IRES in a variety of cellular mRNAs, and many cellular IRES elements were shown to be *bona fide* regulatory elements that direct translation of their respective proteins in a cap- and/or eIF4E-independent manner during various cellular stresses or pathological conditions, such as tumorigenesis, in particular. It is, therefore, not surprising that many of the IRES-harboring mRNAs encode proteins that are key regulators of cell proliferation and survival/apoptosis and their misregulated expression is frequently associated with cancer. Thus, we and others have proposed that the selective regulation of IRES-mediated translation is important for the regulation of cell death and survival (14, 31–34). Indeed, the experimental data from many laboratories have now validated this hypothesis in a number of models [reviewed in Ref. (34, 35)]. The *in vivo* evidence supporting this concept, and particularly in the context of cancer formation, is particularly striking. For example, the selective impairment of IRES-mediated translation but not cap-dependent translation is correlated with enhanced apoptosis of hematopoietic progenitors and stem cells, leading to the fatal progressive bone marrow failure syndrome known as *dyskeratosis congenita* and its associated tumorigenesis (36–38). A switch to IRES-dependent translation has been implicated in breast cancer growth and angiogenic potential *in vivo* (39), and in the acquired resistance of cancer cells to radiation-induced apoptosis (40). Similarly, HPV-induced transformation of human keratinocytes is accompanied by a switch from cap-dependent to IRES-dependent translation (41).

## Mechanisms of eIF2α-Independent Translation

The mechanism of how translation initiation can occur without the eIF2 was first described in viruses that utilize IRES mechanism of translation initiation, and whose translation has been reported to be refractory to reduced availability of ternary complex. The simplest model is the CrPV virus, in which the secondary and tertiary structures of the intergenic region IRES mimic the function of a peptidyl tRNA (Met-tRNA_i_) in the P site, recruit 40S and 60S ribosomal subunits to form 80S, and direct the synthesis of viral polyprotein by inducing ribosomal translocation (42). Hepatitis C virus (HCV) and classical swine fewer virus (CSFV) were both shown to use eIF5B instead of eIF2 to deliver initiator tRNA into the P site of the ribosome (43–45). The canonical role of eIF5B is in ribosome subunit joining, and it does not normally promote binding of initiator tRNA to the 40S subunit. However, it appears to “take over” when eIF2 is inactivated due to phosphorylation. In addition to eIF5B, other cellular proteins including eIF2D (formerly called Ligatin) and MCT-1/DENR were all reported to be able to deliver tRNA into the P site of the ribosome during HCV IRES-mediated translation (46, 47). Their role in supporting cellular mRNA translation during stress has not yet been fully elucidated.

Evidence is starting to emerge that a similar eIF5B-dependent mechanism operates on cellular mRNAs as well. We have investigated the molecular mechanism of eIF2α-independent translation of XIAP mRNA (21). XIAP protein is encoded by two mRNA splice variants that differ only in their 5′ UTR regions. The more abundant, shorter transcript produces the majority of XIAP protein under normal growth conditions by cap-dependent translation. However, during cellular stress, the longer transcript, containing the IRES element, directs efficient translation despite attenuation of global, cap-dependent translation (20). During normal proliferative conditions, when the ternary complex is available in abundance, XIAP translation continues in an eIF2α-dependent mode, similar to other cellular mRNAs. However, upon serum deprivation, the XIAP IRES-dependent translation switches to an alternative, eIF5B-dependent mode to circumvent attenuation due to eIF2α phosphorylation. The ability of the cell to evade ternary complex requirement suggests that cells have evolved an alternative, eIF2α-independent mechanism of tRNA delivery to support a “rescue” mechanism of translation of critical survival proteins under conditions when the “normal” mechanism is not available. Interestingly, a limited investigation of other cellular IRES-containing mRNAs (Bcl-xL, cIAP1, Apaf-1, and p97/DAP5) suggests that not all cellular IRESs utilize eIF5B-dependent mode of tRNA delivery during serum deprivation (21). However, the full spectrum of eIF5B-dependent cellular mRNA transcripts still needs to be determined.

Multiple copies in T-cell lymphoma-1 (MCT-1; also called MCST1, malignant T-cell amplified sequence 1) is an oncogene, which is also implicated in the regulation of cellular mRNA translation, although the precise mechanism of this control is not clear (48). MCT-1 recruits DENR (density regulated protein) to aid in the post-termination ribosome recycling (49). In addition, DENR/MCT-1 complex interacts with the cap complex and enhances translation of a subset of mRNAs whose products are involved in the regulation of apoptosis and the cell cycle. Of note, some of these mRNAs, such as cyclin D1, HSP70, and cIAP1, were previously reported to be translated by an IRES-dependent mechanism (22, 50, 51). Recently, a different cohort of specific mRNAs that require DENR/MCT-1 complex for efficient translation was identified by polysome profiling experiments in DENR-knockdown *Drosophila* cells (48). Interestingly, these mRNAs, enriched for transcriptional regulators and oncogenic kinases, were characterized by the presence of uORFs with strong Kozak sequence. However, further experiments revealed that DENR/MCT-1 promotes reinitiation of translation on these mRNAs, so the mechanism of translation upregulation by DENR/MCT-1 is distinct from the classical uORF-dependent mode that is used by ATF4. Whether or not DENR/MCT-1 can also function in an alternative ternary complex formation in order to deliver initiator tRNA under conditions of cell stress has not been tested.

## eIF2α-Independent Translation and Cancer

Phosphorylation of eIF2α serves to integrate diverse stress signals, both intra- and extracellular, and for both physiologic and pathophysiologic conditions. The role of eIF2α in tumorigenesis has been recently summarized (3). Studies in mouse models and human tumors suggest that targeted disruption of eIF2α phosphorylation could be exploited to increase the efficacy of therapeutic intervention. In addition, initiation of apoptosis (the main mechanism of action of chemotheraphy drugs) results in the caspase-mediated cleavage of eIF2α (52–54), suggesting that inhibition of the ternary complex formation may be part of the translational reprograming accompanying apoptosis.

It is, therefore, important to explore the possibility that tumor cells could gain a selective growth advantage and evade apoptosis by utilizing an eIF2α-independent mode of translation initiation of cancer-related mRNAs, such as XIAP, cIAP1, cyclin D1, and others. As noted above, MCT-1 is an oncogene involved in the regulation of cell cycle and apoptosis. Expression of MCT-1 transforms NIH3T3 and MCF-10A cells (55, 56) and promotes aggressive growth, angiogenesis, tissue invasiveness, and inhibition of apoptosis in breast (57) and lymphoma (58) tumor models. Furthermore, high expression of MCT-1 was reported in 83.9% of human lung carcionomas (59). MCT-1 acts in conjunction with DENR; however, there is no information available on the expression and role of DENR in tumorigenesis yet. A quick survey of the antibody staining data in The Human Protein Atlas^1^ showed that DENR is detected in 74% of the cancers, but the discordantly high expression relative to normal tissue is most noticeable in gliomas, lymphomas, melanoma, skin, and urothelial cancers. Of note, expression of MCT-1 is also particularly high in glioma, melanoma, and urothelial cancers (see text footnote 1), suggesting that the concomitant expression of DENR and MCT-1 in these particular cancers may lead to translational activation of specific mRNAs. This notion, however, awaits experimental validation.

There is no available literature on the possible involvement of eIF2D in cancer with the exception of eIF2D being identified as a hepatocellular carcinoma (HCC) – associated antigen in a study of four HCC patients (60). A survey of The Human Protein Atlas shows increased expression in some head and neck, lymphoma, melanoma, ovarian, pancreatic, stomach, and urothelial cancers (see text footnote 1). Interestingly, eIF2D belongs to a family of PUA (pseudouridine synthase and archaeosine transglycosylase) domain containing proteins, along with DENR and MCT-1 (61). Another notable member of this family is dyskerin (DKC1), which has been shown to play a key role in enabling translation of cancer-related IRES-containing mRNAs, such as Bcl-xL, XIAP (36), p27 (38), and p53 (37). DKC1 deficiency results in the decrease in modification of ribosomal RNA uridines, which consequently renders the ribosome unable to efficiently engage select mRNAs for translation (62). An intriguing possibility, which ought to be experimentally investigated is whether, like DENR/MCT-1 or eIF2D, DKC1 can also participate in the formation of an alternative ternary complex for these mRNAs.

eIF5B was shown to control cell cycle transition and developmental stages during oocytes maturation, by inhibiting cell cycle arrest (63). Interestingly, this study also demonstrated that both the levels of eIF5B and the association of eIF5B with Met-tRNA_i_ were increased during serum starvation of oocytes, ES cells, or in proliferating T cells. This was accompanied by increased phosphorylation of eIF2α, suggesting that under these conditions eIF5B may preferentially enhance translation of specific mRNAs. In addition, depletion of eIF5B impaired translation in mature, but not proliferating cells, further supporting a stage-specific role for eIF5B. Given the essential role of eIF5B in general translation, it is expressed in all normal tissues; however, there is an enhanced expression in some melanoma and ovarian cancers (see text footnote 1). Of note, the majority of the surveyed cancers show reduced eIF5B staining, when compared to the expression of eIF5B in normal tissues, suggesting that a relationship between eIF5B and tumorigenesis is likely not straightforward.

## Future Perspectives

**Figure 2:**
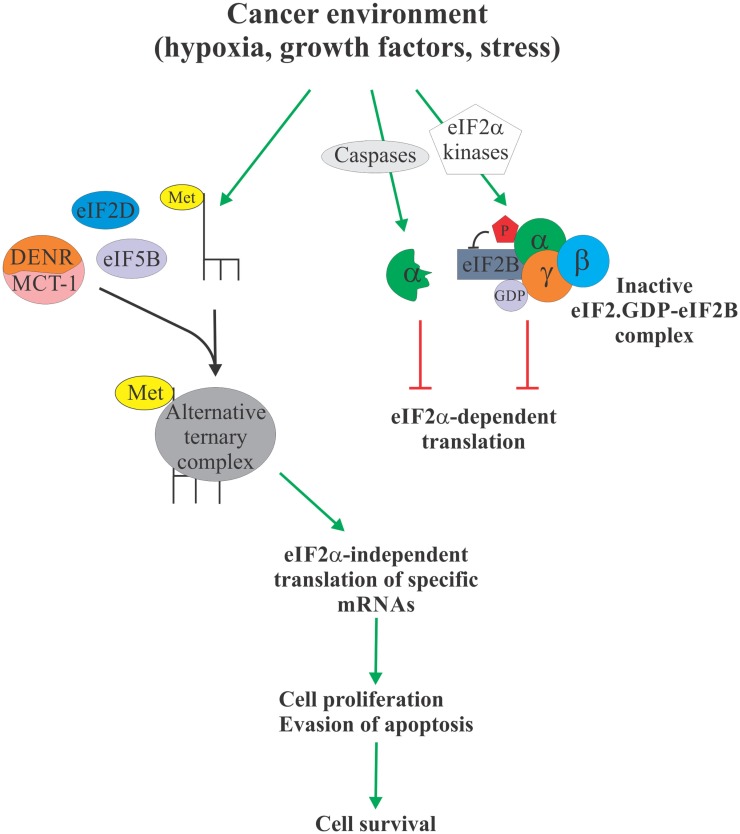
**eIF2α-independent translation in cancer**. Cancer environment, such as hypoxia, growth factor and cytokine signaling, and various forms of stress, leads to enhanced phosphorylation or proteolytic degradation of eIF2α which causes attenuation of protein synthesis due to unavailability of the ternary complex (right). However, this allows Met-tRNA_i_ to form an alternative ternary complex with proteins like eIF5B, eIF2D, and DENR/MCT-1 (left). Alternative ternary complex supports translation of select mRNAs, including those regulating apoptosis and cell cycle progression, in an eIF2α-independent manner, ultimately providing the cancer cell with a survival advantage. Specific targeting of the eIF2α-independent translation mechanism therefore offers a possible therapeutic target to treat cancer.

Over the past two decades, eIF2α has emerged as a critical nexus integrating diverse cellular and extracellular signals to control protein synthesis. More recently, the evidence is mounting to link eIF2α phosphorylation to tumorigenesis [reviewed in Ref. (3)]. In particular, elevated levels of eIF2α phosphorylation were shown to correlate with cancer cell survival. These observations are somewhat paradoxical, since cancer cells typically display elevated protein synthesis levels (64), which would not be compatible with elevated levels of eIF2α phosphorylation. Phosphorylation of eIF2α, therefore, likely serves to activate specific survival pathways, some of which may rely on the formation of an alternative, eIF2α-independent ternary complexes to promote translation of cancer-specific mRNAs (Figure 2). These alternative pathways and their components, such as eIF5B, eIF2D, and DENR/MCT-1, could, therefore, represent as of yet unexplored Achilles heels of cancer. For example, we have shown that eIF5B promotes IRES-dependent translation of an inhibitor of apoptosis XIAP (21). Chemoresistance in several types of cancer cells has been associated with increased expression of XIAP (65–69). Importantly, XIAP has been validated as a potent therapeutic target, and various anti-XIAP strategies for cancer treatment are currently being tested in clinical trials (70). As with many other tumor-specific targets, the specificity of the therapeutics intervention for tumor cells is desirable, since XIAP deficiency in non-cancerous cells can lead to disorders, such as X-linked lymphoproliferative disease (71, 72). Specific targeting of the eIF2α-independent mode of translation of XIAP and other cancer-related mRNAs may provide this needed specificity.

## Author Contributions

MH conceived and wrote the manuscript.

## Conflict of Interest Statement

The author declares that the research was conducted in the absence of any commercial or financial relationships that could be construed as a potential conflict of interest.

## Footnotes

^1^http://www.proteinatlas.org
